# *LoSWEET14*, a Sugar Transporter in Lily, Is Regulated by Transcription Factor *LoABF2* to Participate in the ABA Signaling Pathway and Enhance Tolerance to Multiple Abiotic Stresses in Tobacco

**DOI:** 10.3390/ijms232315093

**Published:** 2022-12-01

**Authors:** Zhen Zeng, Tong Lyu, Yingmin Lyu

**Affiliations:** 1 Beijing Key Laboratory of Ornamental Plants Germplasm Innovation and Molecular Breeding, China National Engineering Research Center for Floriculture, College of Landscape Architecture, Beijing Forestry University, Beijing 100083, China; 2 Beijing Flower Engineering Technology Research Center, Plant Institute, China National Botanical Garden North Park, Beijing 100093, China

**Keywords:** SWEET, sugar transporter, ABRE/ABF, abiotic stresses, lily

## Abstract

Sugar transport and distribution plays an important role in lily bulb development and resistance to abiotic stresses. In this study, a member of the Sugar Will Eventually be Exported Transporters (*SWEET*) gene family, *LoSWEET14*, from Oriental hybrid lily ‘Sorbonne’ was identified. LoSWEET14 encodes a protein of 278 amino acids and is capable of transporting sucrose and some types of hexoses. The transcript level of the *LoSWEET14* gene was significantly increased under various stress conditions including drought, cold, salt stresses, and abscisic acid (ABA) treatment. Overexpression of *LoSWEET14* in tobacco (*Nicotiana tabacum*) showed that the transgenic lines had larger leaves, accumulated more soluble sugars, and were more resistant to drought, cold, and salt stresses, while becoming more sensitive to ABA compared with wild-type lines. Promoter analysis revealed that multiple stress-related cis-acting elements were found in the promoter of *LoSWEET14*. According to the distribution of cis-acting elements, different lengths of 5′-deletion fragments were constructed and the *LoSWEET14-pro3*(*-540 bp*) was found to be able to drive *GUS* gene expression in response to abiotic stresses and ABA treatment. Furthermore, a yeast one hybrid (Y1H) assay proved that the AREB/ABF (ABRE-binding protein/ABRE-binding factor) from lilies (*LoABF2*) could bind to the promoter of *LoSWEET14*. These findings indicated that LoSWEET14 is induced by LoABF2 to participate in the ABA signaling pathway to promote soluble sugar accumulation in response to multiple abiotic stresses.

## 1. Introduction

Plants are exposed to multiple abiotic stresses during growth and development, including drought, extreme temperatures, and high salinity [1]. In response to these stressful environments, plants will undergo physiological, cellular, and molecular adjustments [2], and accumulate more sugars to maintain stable cellular osmotic pressure, thereby improving their resilience to stress and maintaining normal growth [3,4]. Therefore, in addition to providing plants with energy necessary for growth and development, sugars can also be involved in regulating responses to stresses such as drought and low temperature. [5,6].

In higher plants, sugars, especially sucrose, act as the final product of photosynthesis, transported from mature leaves (source) to non-photosynthetic cells (sink) through phloem sieve elements (SE). There are two pathways for loading and unloading sucrose in the phloem, one is the symplastic pathway, mediated by plasmodesmata, and the other one is the apoplastic pathway, which is linked by specific sugar transporters (STs) [7,8]. After being transported to sink tissues, a part of the sucrose is converted into fructose, glucose, or Uridine diphosphade glucose (UDP-glucose) by extracellular invertase and then transported into parenchyma cells by hexose transporters. On the other hand, sucrose that is not hydrolyzed will be stored in the vacuole directly by sucrose transporters [9,10]. Therefore, STs play an important role in the regulation of sugar accumulation.

Sucrose transporters (SUTs) and SWEETs are major transporters for sucrose transported through the apoplastic pathway [7,8,11]. SUT is a type of H^+^-dependent sugar transporter, which needs to be coupled with protons to generate dynamical potential and thus drive the transport of sucrose, whereas SWEET does not require energy to complete the bidirectional transport of sucrose [12,13]. SWEET proteins usually have seven transmembrane domains (TMDs) and are formed by two MtN3 motifs [14,15]. Based on phylogenetic analysis and their functions in sugar transport, SWEET transporters are divided into four clades: clade I and clade II mainly transport hexose, clade III preferentially transports sucrose, while clade IV is responsible for fructose transport [15,16,17].

SWEETs have been shown to play roles in improving fruit quality by altering sugar accumulation in sink organs. Overexpression of *VvSWEET10* in grape (*Vitis vinifera*) callus and tomato (*Solanum lycopersium*) significantly increased the content of glucose, fructose, and total sugar [18]. Elimination of *SlSWEET15* function via (CRISPRs)/CRISPR editing significantly reduced fruit size and weight in tomato [19], while silencing *SlSWEET7a* or *SlSWEET14* instead led to an increase in sugar content in ripe fruits [20]. SWEETs are also widely involved in plant reproductive growth. Mutants of both maize (*Zea mays* L.) *ZmSWEET4c* and its rice (*Oryza sativa*) homolog, *OsSWEET4*, were defective in seed filling [21], while elimination of *SlSWEET15* resulted in severe defects in seed filling and embryo development [19]. In soybean (*Glycine max* L.), *GmSWEET15* could mediate the export of sucrose from the endosperm to the embryo in early seed development [22]. SWEET9 in various plants affects nectar secretion [23].

SWEET genes have also been identified to participate in resistance to abiotic stresses. In *Arabidopsis thaliana*, disruption of *AtSWEET11* and *AtSWEET12* affected plant resistance to cold and frost [24]. At low temperatures, the expression level of *AtSWEET16* was downregulated and the *AtSWEET16* overexpression lines could not accumulate fructose normally [17]. *AtSWEET15*/SAG29 was also induced by low temperature, high salt, and drought stress [25]. Under drought conditions, *MdSWEET17* transgenic lines had higher tolerance to drought and accumulated more soluble sugars, especially fructose [26]. Overexpression of *DsSWEET17* and *DsSWEET12* from *Dianthus spiculifolius* in *Arabidopsis* showed higher tolerance to osmotic and oxidative stresses [27,28]. These findings suggest that *SWEET* genes may be able to receive abiotic stress signals to improve tolerance to adversity by regulating sugar transport and distribution, but the exact signals that induce SWEET have not been fully studied.

The phytohormone abscisic acid (ABA) is known as a key signal for plants to respond to abiotic stress [29,30]. Abiotic stress signals increase ABA levels in plants by activating the expression of genes involved in ABA synthesis, which in turn activates the expression of ABA-responsive genes through ABA-responsive elements (ABREs) in promoters [31]. An increasing number of ABA-regulated genes, such as *ABF*, *CBL9*, and *CIPK3*, have been identified in enhancing plant tolerance to abiotic stress [32]. The basic leucine zipper (bZIP) family is considered as one of the major regulators of stresses, and AREB/ABF (ABRE-binding protein/ABRE-binding factor), a member of bZIP family, plays a universal role in abiotic stress and the ABA signaling pathway [33]. On the other hand, ABA is found to be strongly associated with sugar signals [34]. The *Arabidopsis* ABI3 (ABA Insensitive3) mutant was not sensitive to sugar during germination and showed sugar insensitivity (sis) and sucrose uncoupling (sun) phenotypes [35]. In rice, sucrose plus ABA can improve the source-sink relationship through sucrose and trehalose metabolism in grains, thereby increasing rice yield and quality [36]. Considering the synergistic effects between ABA and sugar, together with their functions in abiotic stress, it is reasonable to speculate that ABA signaling may regulate sugar transport and distribution. This conjecture has been confirmed by recent studies that show that some STs are regulated by bZIP TFs. OsbZIP72 can directly bind to the OsSWEET13 and OsSWEET15 promoters and activate their expression under drought and salt stress [37]. MdAREB2, an ABA-responsive TF in apple (*Malus domestica*), promoted soluble sugar accumulation by activating the expression of *MdSUT2*, and overexpression lines of MdAREB2 also accumulated more soluble sugar [38]. These researches suggest that SWEETs may be involved in ABA signaling to alter the sugar transport and distribution process under abiotic stress.

Lily (Lilium spp.) is one of the most important bulbous flowers worldwide. Bulb vernalization and development plays a crucial role in the development of the lily industry and has been proved to be closely related to carbohydrate metabolism [10,39]. In our previous study, it has been found that sugar transporter- and metabolism-related genes were extensively involved in sugar distribution processes to affect the sink-source transformation of bulbs, and were induced by various abiotic stresses [40]. These findings indicate that during vernalization, which is also a kind of abiotic stress limiting the outdoor cultivation of lilies, sugars accumulated in bulbs may act as signals to activate bulbs to break dormancy. However, the specific regulatory mechanism of sugar transport in response to abiotic stress is still unknown. In the present study, a gene of STs, *LoSWEET14*, was cloned from oriental *Lilium* ‘Sorbonne’, and was found to be induced by drought, cold, salt treatments, and exogenous ABA. In *LoSWEET14* transgenic tobacco (*Nicotiana tabacum*), more starch, sucrose, and soluble sugar were accumulated, and the transgenic plants showed higher tolerance to low temperature, drought, and high salt stresses and were more sensitive to ABA. In addition, the ABA-binding factor of lilies (*LoABF2*) could bind directly to ABREs on the *LoSWEET14* promoter. These findings suggest that LoSWEET14 may be involved in the ABA signaling pathway to regulate sugar accumulation under abiotic stresses, which provides a new idea for improving the quality of lily bulbs to respond to abiotic stresses including cold vernalization.

## 2. Results

### 2.1. Molecular Cloning and Sequence Analysis of LoSWEET14 Gene

**Figure 1:**
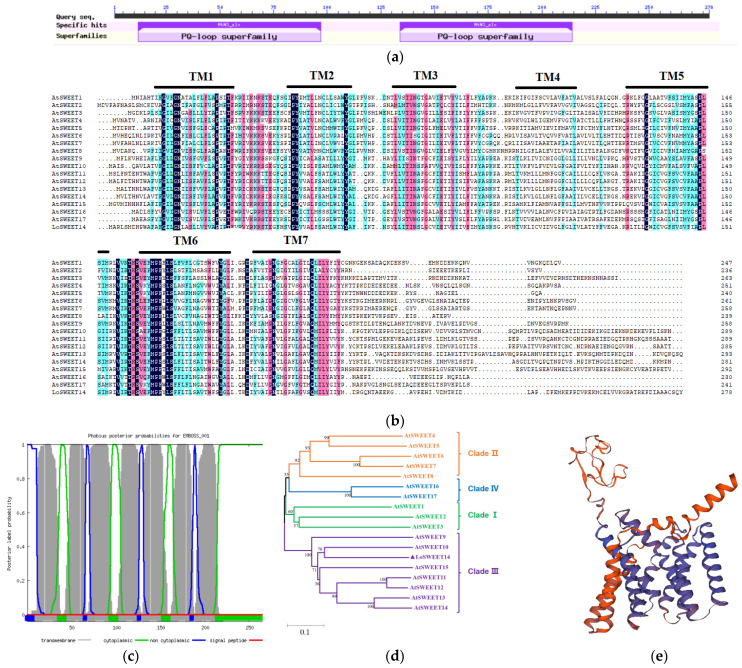
Characterization of LoSWEET14 protein. (**a**) Conserved domain analysis of *LoSWEET14* on NCBI. (**b**) Multiple sequence alignment of LoSWEET14 with *Arabidopsis thaliana* (AtSWEET1 to AtSWEET17). The conserved domain is marked in color and identical amino acids are shaded in black. The seven TM domains are marked out with black lines. (**c**) Putative transmembrane domains of LoSWEET14 using Phobius. (**d**) Phylogenetic analysis of LoSWEET14 with *Arabidopsis* SWEETs constructed by MEGA11.0 software. (**e**) The tertiary structure of LoSWEET14 protein on SWISS-MODEL.

The cDNA sequence of *LoSWEET14* was found based on the previous transcriptome data of our laboratory [41]. The open reading frame (ORF) of *LoSWEET14* was 837 bp (the full-length is shown in Figure S1). It encodes a protein of 278 amino acids, with a predicted molecular weight of 31.37 kDa, and an isoelectric point (*p*I) of 8.49. The conserved domain sequence (CDS) analysis of *LoSWEET14* on NCBI revealed that it contained two MtN3 domains (Figure 1a), and the multiple sequence alignment showed that it contained seven TMDs (Figure 1b), which was consistent with the protein structure prediction on Phobius (Figure 1c). A phylogenetic tree using SWEET sequences in *Arabidopsis thaliana* according to the NJ method was constructed, which showed that *LoSWEET14* was closest to *AtSWEET10* in homology (Figure 1d), and both belonged to Clade III of the SWEET family [15]. The protein tertiary structure model on SWISS-MODEL showed that the protein is mainly composed of α-helixes and random helixes (Figure 1e), and these features were consistent with previous studies [14].

### 2.2. Sugar Transport Activities in the Yeast EBY.VW4000 of LoSWEET14

**Figure 2:**
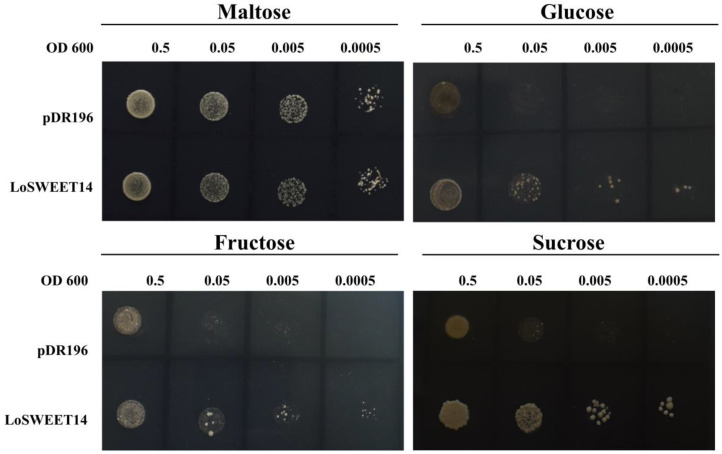
Sugar transport ability of LoSWEET14. Growth of blank pDR196 and LoSWEET14-pDR196 transformed yeast on the SD/−Ura media supplemented with 2% maltose, 2% glucose, 2% fructose, and 2% sucrose.

In order to confirm the sugar transport activity of LoSWEET14, the gene was ectopically expressed in yeast strain EBY.VW 4000, which is a specific hexose transporter-deficient strain that lacks 20 endogenous hexose transporters and cannot grow in media with hexose as the main carbon source, but can grow in media with maltose [42]. Results showed that strains transformed with blank vector pDR196 or LoSWEET14-pDR196 were both able to grow in a maltose medium, while in a medium with sucrose as the main carbon source, only strains with LoSWEET14 could obviously recover growth. In media with fructose or glucose as the main carbon source, the yeast strain with LoSWEET14 showed signs of growth, but they were not obvious (Figure 2). These results suggest that LoSWEET14 can transport sucrose and a small number of hexoses such as fructose and glucose.

### 2.3. Expression Patterns of LoSWEET14 Gene under Multiple Abiotic Stresses and Exogenous ABA Treatment

**Figure 3:**
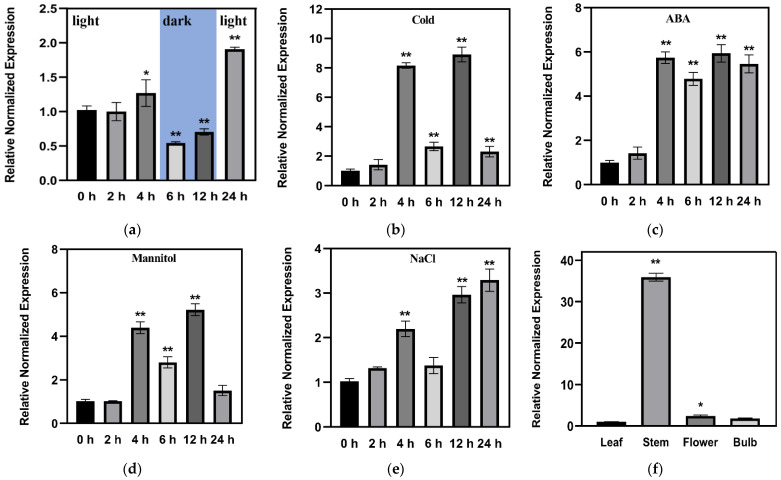
Expression patterns of *LoSWEET14* in leaves under stress treatments and in different tissues. Eight-week-old lily seedlings were untreated, (**a**) were treated with 4 °C (**b**), 150 µM ABA (**c**), 200 mM mannitol (**d**), and 200 mM NaCl (**e**) for 24 h, respectively. Tissue-specific expression analyses were conducted in the leaf, stem, bulb, and flower (**f**). The seedings were under a 14 h light and 10 h dark (from 5 h to 15 h) photoperiod. Each value indicates Mean ± SE (standard error). Asterisks indicate a significant difference ** *p* < 0.01 and * *p* < 0.05 compared with the corresponding controls.

To explore the possible response of LoSWEET14 to abiotic stress and ABA, we analyzed the expression pattern of the *LoSWEET14* gene in a lily treated with cold, mannitol, NaCl, and ABA by qRT-PCR. Results showed that the transcript level of *LoSWEET14* was rapidly and significantly upregulated within 4 h after various treatments and then decreased after 6 h treatment of low temperature, drought, and high salt conditions (but was still higher than that of before treatment), and was specifically induced again after 12 h. After 24 h, the expression level under cold and drought treatment began to return to a normal level (Figure 3b,d), while under salt stress it continued to increase (Figure 3e). Under ABA treatment, the expression level of *LoSWEET14* continued to be significantly upregulated to 24 h, with about 5-fold increase (Figure 3c). Interestingly, expression levels of *LoSWEET14* under various treatments for 6 h were systematically lower. To clarify whether expression of the *LoSWEET14* gene was affected by light/dark transition, the expression pattern of *LoSWEET14* in a mock plant during the 24 h of this experiment was also analyzed (Figure 3a). Results showed that when the light switched off (from 5 h to 15 h), expression levels of *LoSWEET14* (6 h and 12 h) were significantly decreased, on the other hand, when the light switched on, expression levels of *LoSWEET14* were relatively higher, indicating that *LoSWEET14* might be regulated by photoperiod. Despite the influence of light/dark transition, the expression levels of *LoSWEET14* were also induced by cold, mannitol, NaCl, and ABA apparently. These findings indicate that LoSWEET14 plays a role in abiotic stresses and ABA signaling. In addition, tissue-specific expression analysis revealed that *LoSWEET14* was significantly higher expressed in the stem, followed by the flower, while being relatively lower in the bulb and leaf (Figure 3e).

### 2.4. Overexpression of LoSWEET14 Improved Cold and Drought Tolerance in Tobacco

**Figure 4:**
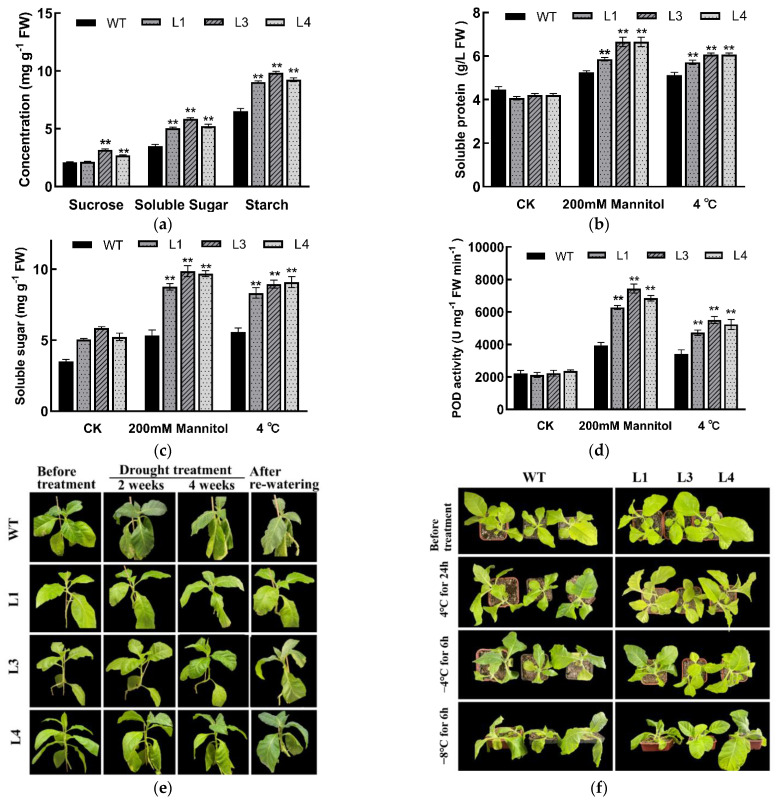
Overexpression of the *LoSWEET14* gene increased sugar content and improved the resistance to drought and cold stress of *Nicotiana tabacum*. Sugar content of transgenic and WT plants (**a**). Analysis of soluble protein (**b**), soluble sugar (**c**), and POD activity (**d**) in WT and transgenic lines untreated (CK), treated with 200mM mannitol, 4 °C, respectively. Morphological changes of WT and transgenic tobacco under drought (**e**) and low temperature (**f**) stress. Each value indicates Mean ± SE (standard error). Asterisks indicate a significant difference ** *p* < 0.01 compared with the corresponding controls.

To further investigate functions of the LoSWEET14 protein, transgenic tobacco plants that overexpressed *LoSWEET14* were generated. Three transgenic lines (L1, L3, and L4) with relatively higher expression were selected for subsequent study (Figure S2). Compared with wild type (WT), the leave size of the transgenic plants was obviously larger (Figure S3), and the contents of starch, sucrose, and soluble sugar in the leaves were significantly higher (Figure 4a), indicating that LoSWEET14 can affect the process of sugar accumulation and allocation in tobacco.

In order to determine whether LoSWEET14 is involved in resistance to abiotic stresses, both the *LoSWEET14* transgenic lines and WT plants were treated with multiple stresses simultaneously. For drought stress, after 4 weeks of watering cessation, the WT plants showed obvious drought damages, with leaves that were shriveled and wilted. After 7 days of rewatering, these damages could not be relieved, and plants were in a state of complete wilting and death. The transgenic lines, on the other hand, showed only slight leaf crinkling and recovered quickly after rewatering (Figure 4e). In addition, after treatment with 200 mM mannitol for 24 h, the transgenic lines accumulated more soluble protein and soluble sugar compared with WT plants (Figure 4b,c), and the activity of peroxidase (POD) was also significantly higher (Figure 4d).

As for cold stress, WT and transgenic plants were exposed to 4 °C for 24 h, followed by −4 °C for 6 h, and −8 °C for 6 h. We found that treated at 4 °C for 24 h, leaves of WT plants turned greener, and after treatment at −8 °C, WT plants could no longer maintain upright morphology and almost could not survive. However, the transgenic tobacco plants hardly changed in morphology treated at −4 °C for 6 h, with only slight leaf shrinkage after treatment at −8 °C (Figure 4f). Similarly, the transgenic lines accumulated more soluble protein and soluble sugar compared to WT plants after cold (Figure 4b,c), and the POD activity also increased significantly (Figure 4d). These results suggest that the *LoSWEET14* gene can improve the resistance of tobacco to drought and cold stress.

### 2.5. Overexpression of LoSWEET14 Increased Tolerance to NaCl and Sensitivity to ABA of Tobacco Germinating Seeds

**Figure 5:**
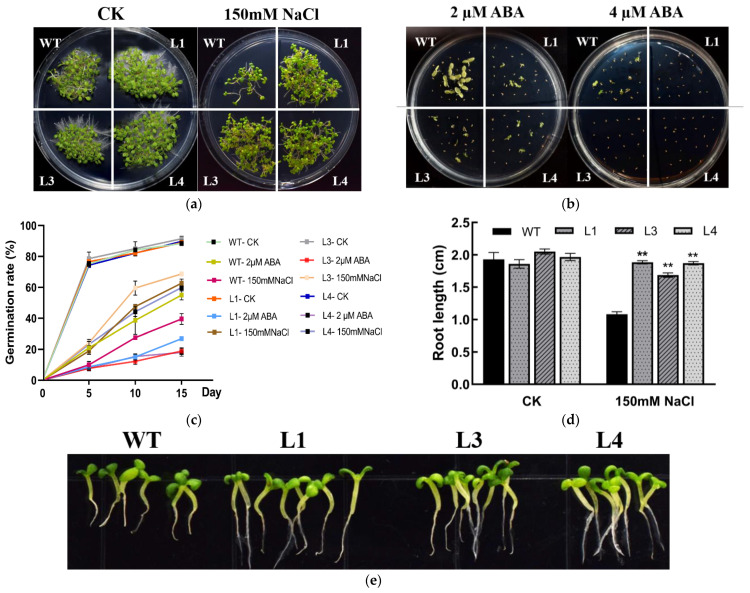
Overexpression of the *LoSWEET14* gene improved salt stress resistance and ABA sensitivity of *Nicotiana tabacum*. WT and *LoSWEET14* transgenic tobacco were sown on MS media containing 0 µM ABA/NaCl (CK), 150 mM NaCl (**a**), 2 µM ABA, 4 µM ABA (**b**), respectively. Germination rate of WT and transgenic tobacco seeds under different treatments (**c**), root length (**d**) and growth (**e**) of WT and transgenic tobacco treated with 150 mM NaCl were observed and measured 2 weeks after germination. Each value indicates Mean ± SE (standard error). Asterisks indicate a significant difference ** *p* < 0.01 compared with the corresponding controls.

The response of LoSWEET14 to salt stress and ABA stimulation were also investigated. In a normal MS medium, the germination rates of WT and transgenic seeds were both about 90% without significant difference (Figure 5c). However, in an MS medium supplemented with 150 mM NaCl, the germination rate of WT lines decreased significantly to less than 40%, while the germination rate of the transgenic tobacco was about 60–70%, significantly higher than that of WT under NaCl treatment (Figure 5a,c). Furthermore, the root length of WT was significantly shorter compared with the overexpression lines (Figure 5e). In addition, WT and transgenic lines were seeded in an MS medium containing 2 µM ABA and 4 µM ABA, respectively. In the medium containing 2 µM ABA, the germination rate of the WT lines was about 55%, while only about 20% of the transgenic lines were able to germinate (Figure 5b,c). Additionally, in the medium containing 4 µM ABA, a small percentage of WT seeds could still survive while the transgenic seeds could barely germinate (Figure 5b). These findings suggest that the *LoSWEET14* gene can improve resistance to salt stress and sensitivity to ABA in tobacco seeds.

### 2.6. Overexpression of LoSWEET14 Induced the Expression of Stress-Related Genes in Tobacco

**Figure 6:**
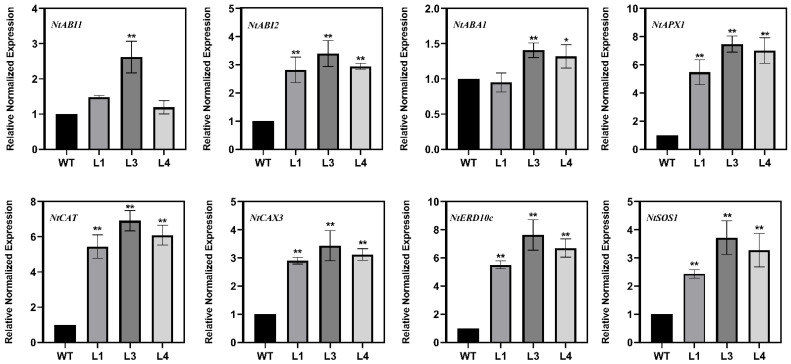
Expression analysis of stress related genes in 4-week-old seedlings of WT and *LoSWEET14* transgenic tobacco leaves under normal condition. Each value indicates Mean ± SE (standard error). Asterisks indicate a significant difference ** *p* < 0.01 and * *p* < 0.05 compared with the corresponding controls.

It has been demonstrated that *LoSWEET14* transgenic plants can respond to abiotic stress and ABA signaling morphologically and physiologically as mentioned above. Then, the expression levels of stress and ABA response-related genes in transgenic and WT tobacco were determined. Results showed that the relative expression levels of genes including *NtABI2*, *NtAPX1*, *NtCAT*, *NtCAX3*, *NtERD10c*, and *NtSOS1* were significantly higher in all three transgenic lines than in WT. *NtABI1* was significantly expressed in the L3 strain, and *NtABA1* was significantly expressed in the L3 and L4 strains (Figure 6). These findings suggest that overexpression of *LoSWEET14* may enhance plant stress resistance by increasing the expression of stress response-related genes.

### 2.7. Promoter Analysis of SWEET under Abiotic Stress

**Figure 7:**
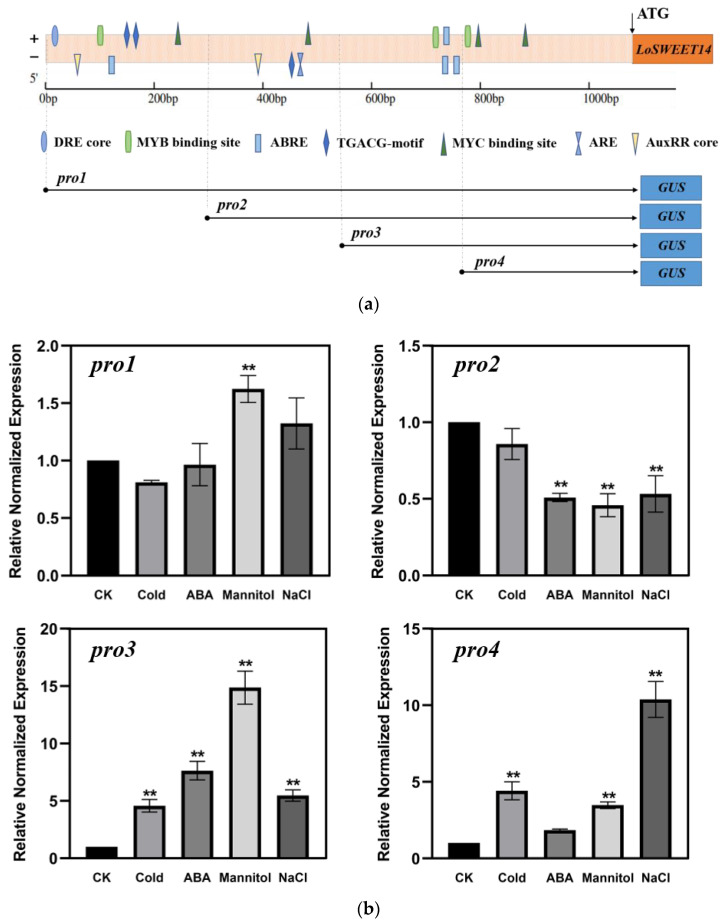
Analysis of the *LoSWEET14* promoter in transgenic tobacco under stress. (**a**) Distribution of major stress-related cis-acting elements in the promoter region of *LoSWEET14* and schematic diagram of truncated *LoSWEET14*-pro fused GUS constructs. The position of the start codon is defined as +1. Different cis-acting elements are represented by different shapes. The numbers on the left represent the length of the 5′ truncated fragments. (**b**) The expression levels of *GUS* gene in promoter transgenic *Nicotiana tabacum* treated with 4 °C, 50 µM ABA, 200 mM NaCl, and 200 mM Mannitol. Each value indicates Mean ± SE (standard error). Asterisks indicate a significant difference ** *p* < 0.01 compared with the corresponding controls.

To clarify the stress-induced regulatory mechanism of LoSWEET14, a 1106 bp upstream of the ATG sequence of its promoter was cloned (Figure S4), of which a variety of cis-acting elements related to abiotic stresses and hormones were predicted, including DRE core, MYB binding site, TGACG motif, MYC binding site, ARE motif, and AuxRR-core. It is also worth noting that there are three ABREs in the promoter region (Table S1, Figure 7a). To further understand the response of the promoter to adversity, in addition to the full length of the promoter *pro1* (−1107 bp), three 5′-truncated fragments, *pro2* (-807 bp), *pro3* (−540 bp), and *pro4* (−357 bp), were constructed, based on the position of the ABRE elements and distribution of other stress-related cis-acting elements (Figure 7a), and were cloned into the pBI121-GUS vector to replace the CaMV35S promoter to drive *GUS* expression in tobacco, respectively.

Results showed that under normal growth conditions, GUS staining could be detected in transgenic plants with different lengths of the promoter, while it was not as strong as in CaMV35S transgenic lines (Figure S5), indicating the weak activity of the *LoSWEET14* promoter under normal conditions. After cold, ABA, mannitol, or NaCl treatment, no visible GUS staining was observed in *pro2*-transgenic lines, and *pro1*-transgenic lines could be stained only under mannitol treatment (Figure S5). In contrast, *pro3* and *pro4*-transgenic lines were all stained after various treatments. Furthermore, in *pro3*-transgenic lines, GUS staining increased significantly in response to cold stress, as well as ABA and mannitol treatments. In *pro4*-transgenic lines, GUS staining increased significantly in response to cold stress and NaCl treatment (Figure S5). The expression levels of the *GUS* gene driven by these promoter fragments under different stresses were also analyzed. In *pro1*-transgenic lines, expression of *GUS* only increased significantly under mannitol treatment. The expression level of *GUS* in *pro2*-transgenic lines treated with ABA, mannitol, or NaCl even decreased significantly. On the other hand, in *pro3* and *pro4*-transgenic lines, the transcript level of *GUS* increased significantly under various stress conditions, except for *pro4*-transgenic lines treated with ABA, which may be due to the absence of ABREs in the *pro4* fragment (Figure 7b). Overall, the expression patterns of the *GUS* gene were consistent with the GUS staining analysis in transgenic tobacco. These results indicate that cis-acting elements in *pro3* and *pro4* may play positive regulatory roles in response to abiotic stress.

### 2.8. LoABF2 Can Bind to the Promoter of LoSWEET14

The ABRE-binding protein AREB/ABF belongs to the group A subfamily of the bZIP family and is involved in the ABA response and various other forms of abiotic stress processes [33,43]. Since multiple ABREs were predicted in the *LoSWEET14* promoter sequence (Figure 7a), it prompted us to further investigate whether LoSWEET14 was regulated by AREB/ABF TFs. From the transcriptome data obtained in our previous study [39], two AREB/ABF genes with full-length of ORF were identified and the expression heatmap and Pearson’s correlation coefficients showed that the expression patterns of *LoABF2* and *LoSWEET14* were very similar under treatment of exogenous ABA (Figure S6, r = 0.82). Therefore, the Y1H assays were performed to verify the interaction between the *LoABF2* protein (Sequence information of *LoABF2* can be found in Figure S7) and the *LoSWEET14* promoter.

**Figure 8:**
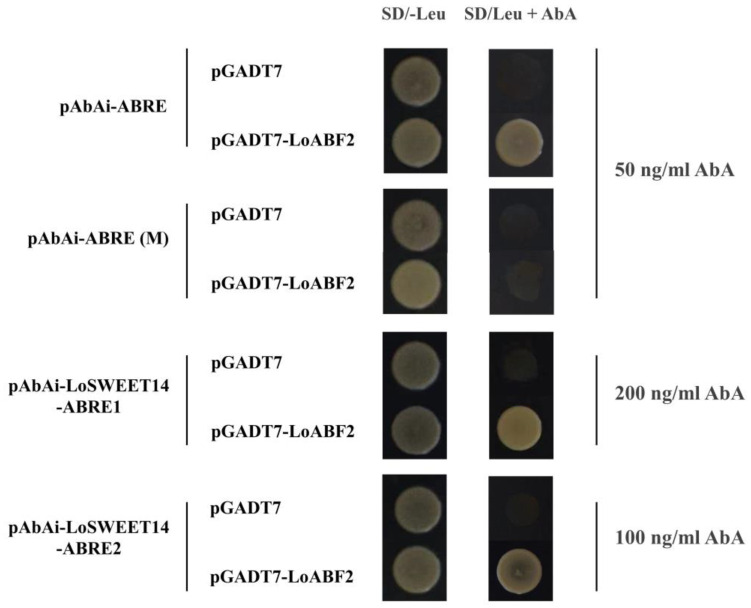
Yeast one-hybrid assay of interaction between *LoABF2* gene and the promoter of *LoSWEET14*. Y1H yeast strains were co-transformed into the prey vector pGADT7-LoABF2 with bait pAbAi-ABRE, pAbAi-ABRE(M), pAbAi-LoSWEET14-ABRE1 and pAbAi-LoSWEET14-ABRE2, respectively, and cultured on SD/−Leu medium containing AbA with corresponding concentration, with pGADT7 empty vector used as the negative control.

When the 1106 bp fragment of the *LoSWEET14* promoter was inserted into the pAbAi vector, the recombinant bait pAbAi-LoSWEET14pro yeast strains could not be inhibited by 900 ng/mL AbA (Aureobasidin A), so the *LoSWEET14* promoter fragments pro-LoSWEET14-ABRE1 (−1103 bp to −1000 bp) containing one ABRE element and pro-LoSWEET14-ABRE2 (−499 bp to −334 bp) containing two ABRE elements (Figure S4) were isolated for further research. The ABRE element and its mutant ABRE(M), fragments LoSWEET14-ABRE1 and LoSWEET14-ABRE2 were cloned into the pAbAi vector, respectively. The minimum inhibitory concentrations of AbA for these recombinant bait yeast strains were 50 ng/mL, 50 ng/mL, 200 ng/mL, and 100 ng/mL, respectively (Figure S8). The *LoABF2* gene was fused to the activation domain (AD) of pGADT7 vector. Yeast strains co-transformed pGADT7-LoABF2 with pAbAi-ABRE, pAbAi-LoSWEET14-ABRE1, or pAbAi-LoSWEET14-ABRE2 could grow well on SD/−Leu plates containing 50 ng/mL, 200 ng/mL, and 100 ng/mL AbA, respectively, while no growth was observed in strains co-transformed pGADT7-LoABF2 with the negative control bait pAbAi-ABRE (M) (Figure 8, whole figures without separations are shown in Figure S8). These results showed that the *LoABF2* transcription factor is involved in the regulation of *LoSWEET14*.

## 3. Discussion

In higher plants, assimilates produced by photosynthesis in mature leaves are transported to sink organs to maintain plant growth and development, mainly in the form of sucrose, which requires the participation of sugar transporters [7,9]. In this study, a sugar transporter, LoSWEET14 from lilies, was cloned and characterized. SWEET proteins typically contain seven TMDs forming two MtN3/Saliva domains [14], which is consistent with the sequence and structure analysis of *LoSWEET14* in our research (Figure 1). In *Arabidopsis*, all *AtSWEETs* can be divided into four clades. Clade I includes *AtSWEET1-3*, and clade II includes *AtSWEET4-8*, which mainly transport hexoses, especially glucose. *AtSWEET9-15* belongs to clade III, which preferentially transport sucrose, and clade IV contains two members, *AtSWEET16* and *AtSWEET17*, which are localized in the vacuolar membrane and mainly mediate the transport of fructose [15,16,17]. Phylogenetic tree revealed that *LoSWEET14* was most closely related to *AtSWEET10* which belongs to clade III and mainly mediates the transport of sucrose (Figure 1). However, analysis of sugar transport activity in our research showed that LoSWEET14 mainly transported sucrose, and could also transport a small amount of glucose and fructose (Figure 2), while the *IbSWEET10* of sweet potato (*Ipomoea batatas* L.), which also belonged to clade III and is closely related to *AtSWEET10*, could only transport sucrose [44]. Such differences suggest that in lilies, SWEET proteins may have a broader substrate binding capacity and participate in more complex biological activities.

Sugar not only provides essential energy for plant growth, but also has a profound effect on cell division and expansion [45]. Soluble sugars including sucrose, glucose, and fructose can stimulate organ proliferation to produce larger and thicker leaves, as well as increase tuber size and the number of adventitious roots [46,47]. In addition, it has been found in recent studies that different sugar signals generated by photosynthesis and carbon metabolism in sink and source tissues play similar roles to plant hormones to modulate plant growth and development, and responses to drought, low temperature, and other stresses [9]. In the process of bulb formation and expansion in lilies, exogenous sucrose not only plays a physiological role, but also stimulates the function of sucrose as a signaling molecule, affecting the activities of sucrose synthase (SuSy) and sucrose phosphate synthase (SPS), resulting in changes to endogenous sucrose sugar metabolism, which ultimately affects bulb formation and expansion [48]. In our previous research, the functions of 16 sugar transporter- and metabolism-related genes in assimilate transport in lilies were analyzed, and the expression of *LoSWEET14* was significantly correlated with starch content in bulbs, and with soluble sugar content in leaves, indicating that LoSWEET14 might play a key role in sink–source relationship change in bulbs [40], but there was no further confirmation. In the present study, we found that *LoSWEET14* transgenic tobacco accumulated more starch, sucrose, and soluble sugars compared with WT plants in leaves (Figure 4a), and the size of transgenic leaves were also significantly larger (Figure S3). Similar phenomena occurred in some other plants. *Arabidopsis* overexpression of *AtSWEET16* strains grew faster and had larger leaves than those of WT lines [17]. *AtSWEET4* transgenic plants were taller and could accumulate more fructose and glucose, while knock-down of *AtSWEET4* caused a reduction in the contents of glucose and fructose and a smaller plant size [49]. However, it was totally opposite in tomato, in that silence of *SlSWEET7a* or *SlSWEET14* led to taller plants and larger fruits (in *SlSWEET7a*-silenced strains) [20]. Overall, these findings suggest that *SWEET* genes can change the external morphological characteristics of plants and affect fruit development by changing the accumulation and distribution process of sugar, which provides a new perspective to improve the quality of bulbs in lilies and fruit quality in production.

In addition to providing energy necessary for growth and development, sugars also play roles in maintaining cell osmotic pressure under abiotic stress and induce the expression of genes related to metabolism and stress resistance [6,9,50]. Previous studies have shown that sugar transporters are involved in plant growth and abiotic stresses [51]. In the present study, the transcript levels of *LoSWEET14* were found to be significantly upregulated under drought, cold, and salt treatments (Figure 3), and *LoSWEET14* transgenic tobacco also proved to be more tolerant to various kinds of abiotic stresses than WT lines at morphological and physiological levels (Figure 4 and Figure 5). In response to abiotic stress, plants have evolved a series of complex enzymatic and non-enzymatic antioxidant defense mechanisms to avoid the production of excessive reactive oxygen species (ROS), which cause serious harm to plant growth and development [52]. Soluble sugars, acting as osmo-protectants, can regulate osmotic pressure and remove toxic ROS in response to stresses [47]. In wheat (*Triticum aestivum* L.) seedlings, it has been reported that low concentrations of glucose could enhance the activity of antioxidant enzymes, such as peroxidase (POD), catalase (CAT), and superoxide dismutase (SOD) [53]. In our research, the *LoSWEET14* transgenic plants not only showed a significant increase in soluble sugar and sucrose content compared with WT lines, but also displayed an obvious upregulation in the transcript levels of antioxidant-related genes such as *NtAPX1*, *NtCAT*, and *NtCAX3* by qRT-PCR analysis (Figure 6). In addition, under low temperature and drought stress, soluble sugar content and POD activity were also significantly increased in the transgenic lines (Figure 4). These findings suggest that LoSWEET14 can affect the expression of antioxidase-related genes by increasing endogenous sugar content, thus reducing the accumulation of ROS in plants under abiotic stresses.

Moreover, it has been revealed in much research that ABA plays a key role in plant resistance to abiotic stress and can be synchronized with sugar signaling [30,45,47]. In lilies, the expression of various sugar metabolism- and transport-related genes were induced by exogenous ABA [40], and in rice it induced the expression of *OsSWEET13* and *OsSWEET15* [37]. *AtSUC2*, *AtSUC4*, and *AtSUC9* of *Arabidopsis* were also upregulated by ABA treatment [32,54]. These findings showed that ABA may be a link between abiotic stress and sugar transport signals. In this study, the transcript level of *LoSWEET14* was also found to be significantly increased under ABA treatment (Figure 3), and the *LoSWEET14* transgenic plants enhanced the sensitivity to ABA stimulation of tobacco seeds (Figure 5). Analysis of the activity of 5′-deletion fragments showed that only *pro3* and *pro4* could significantly activate *GUS* gene expression and make leaves stain under abiotic stress, while *pro1* and *pro2* could not. It can be speculated that elements in *pro3* and *pro4* might play key roles in responses to abiotic stresses. However, *pro4* could not induce *GUS* expression under ABA treatment (Figure 7). This may be due to the difference of cis-acting elements on *pro3* and *pro4* that the *pro4* fragment does not contain ABRE elements, while the fragment of *pro3* is the most enriched region of ABREs. The GUS staining and expression analysis prompted us to speculate that the ABRE element may be an important receiver of the *LoSWEET14* gene to abiotic stress. However, the activity and expression patterns of full-length promotor in lilies under stresses still need further experiments to be confirmed and clarified.

It has been shown that the ABRE-binding protein AREB/ABF, a member of the *bZIP* TF family, could play a general role in both abiotic stress and ABA signaling [33]. In our research, an AREB/ABF TF in lilies (*LoABF2*) was also found to be closely related to *LoSWEET14* under ABA treatment (Figure S6) and could bind to the promoter fragments of *LoSWEET14* directly (Figure 8). These findings suggested that the *LoSWEET14* gene might be regulated by *LoABF2*. The transcriptional regulatory mechanism of SWEET proteins has been reported in previous studies. Rice *DoF11* TF could directly bind to the promoter of *OsSUT1*, *OsSWEET11*, and *OsSWEET14* to regulate the sugar translocation process from source to sink [55]. Recent studies also found that *OsbZIP72* FT can bind to *OsSWEET13* and *OsSWEET15* promoters to regulate sucrose translocation and partitioning under salt and drought stress [37]. On the other hand, the AREB/ABF TF are also inversely regulated by sugar signaling and sugar transports. The expression of *AtbZIP1* was inhibited by glucose in a rapid, sensitive, and reversible manner through the HXK1 signaling pathway [56]. In leaves of *MdSUT2-TRV* transgenic apples, the expression level of *MdAREB2* TF and content of sucrose were both decreased, whereas overexpression of *MdSUT2* in *MdAREB2-TRV* transgenic plants increased sucrose content, indicating that *MdAREB2* promotes sugar accumulation, at least partially through *MdSUT2* [38]. While in our research, whether *LoABF2* can directly be involve in sugar accumulation or partially promote sugar accumulation through STs like *MdAREB2* still needs further investigation.

**Figure 9:**
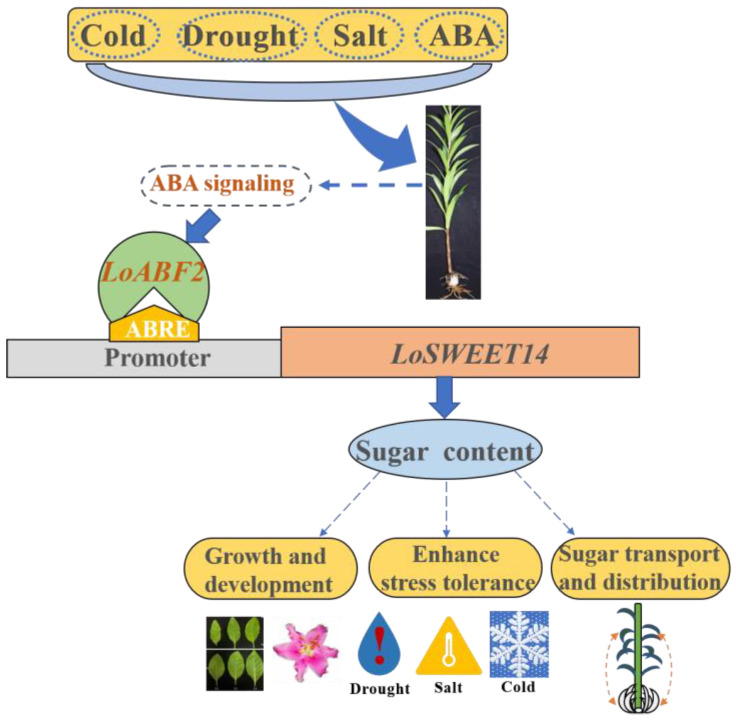
A model of the ABA signal-mediated transcription factor *LoABF2* binding to the promoter of *LoSWEET14* to induce sugar accumulation under abiotic stress.

In conclusion, LoSWEET14 is a sugar transporter that mainly transports sucrose and is induced by various abiotic stresses. Overexpression of *LoSWEET14* in tobacco increased the content of starch, soluble sugar, and sucrose, as well as improved plant resistance to drought, low temperature, and salt stress, and the transgenic plants showed hypersensitivity to ABA. We demonstrated that the *LoABF2* transcription factor can bind to the ABRE elements of the *LoSWEET14* promoter and thus activate the expression of the *LoSWEET14* gene, which can affect sugar transport in bulbs to break dormancy and change the sink-source relationship between underground and aboveground tissues, and furthermore can enhance tolerance to abiotic stresses of lilies (Figure 9). This regulatory mechanism sheds light on the importance of sugar transport promoted by ABA signals and related stresses, thus providing new ideas for cultivation of lily bulbs with higher quality.

## 4. Materials and Methods

### 4.1. Plant Materials

The oriental lily ‘Sorbonne’ was used as the experimental plant material in the present study and it was prepared as described in our previous study [40]. Box planting was carried out under the conditions of 70% relative humidity, 25 °C/18 °C day/night temperature, and 14 h light and 10 h dark photoperiod. For expression analysis of *LoSWEET14* under abiotic stresses, the 8-week-old seedlings were treated with 4 °C, 200 mM NaCl, 200 mM mannitol, and 150 µM ABA for 24 h, respectively. The leaves of the seedlings were collected at 0 h (control), 2 h, 4 h, 6 h, 12 h, and 24 h immediately and all samples were frozen with liquid nitrogen quickly and stored at −80 °C.

The wild type tobacco (*Nicotiana tabacum*) ‘NC89′ was used for transgenic research of *LoSWEET14*. The tobacco plants were grown under conditions of 24 °C/18 °C, 16 h light/8 h dark with 1000lx light intensity, and 65% relative humidity.

### 4.2. Cloning and Sequence Analysis of LoSWEET14

The complete cDNA sequence (Figure S1) of *LoSWEET14* was amplified with primers designed in Table S2 and inserted into the pCloneEZ-Blunt vector (Clone Smarter, USA). The sequenced plasmids were used as templates for subsequent experiments. DNAMAN (version 6) was used for multiple sequence alignment, and the phylogenetic tree was constructed according to the NJ method by MEGA11.0 software. TMDs were predicted on Phobius (https://phobius.sbc.su.se/, accessed on 10 July 2022). The ProtParam tools (https://web.expasy.org/protparam/, accessed on 10 July 2022) were used to predict molecular weight (MW) and theoretical isoelectric point (*p*I). The tertiary structure of the LoSWEET14 protein was predicted on SWISS-MODEL (https://swissmodel.expasy.org/, accessed on 10 July 2022).

### 4.3. RNA Isolation and Quantitative Real-Time PCR Analysis

The total RNAs were extracted using an EASYspin Plus plant RNA rapid extraction kit (Aidlab, Beijing, China). The degradation and contamination concentration of RNA were determined by 1% agarose gel. The obtained RNA was reversely transcribed into cDNA by the PC54-TRUEscript RT kit (+gDNA Eraser) (Aidlab, Beijing, China). A Bio-Rad/CFX Connect™ Real-Time PCR Detection System (Bio-Rad, San Diego, CA, USA) was used to perform qRT-PCR with TB Green^®^ Premix Ex TaqTM II (Takara, Shiga, Japan) according to the protocol. Primers used in qRT-PCR were designed by Primer Premier 5.0, and primer sequences used in this study are shown in Table S2. Each sample was repeated three times, and the relative expression level was calculated using the 2^−ΔΔ^Ct method with *TIP1* as the reference gene in lily and *Ntactin* as the reference gene in tobacco.

### 4.4. Complementation of LoSWEET4 Gene in Yeast Mutants EBY.VW4000

The ORF of *LoSWEET14* without a stop codon was inserted between the *EcoRI* and *SpeI* restriction sites of the pDR196 vector. The pDR196-LoSWEET14 fusion plasmid and the empty vector were transferred into the EBY.VW4000 yeast strain referring to the Quick Easy Yeast Transformation Mix Kit (Clontech, Mountain View, CA, USA), respectively. Transformants were screened in an SD/-Ura plate added with 2% maltose and the transformed cells were cultured in an SD/-Ura liquid medium to OD = 0.5, then it was diluted and spot coated in SD/-Ura plates containing 2% maltose, 2% sucrose, 2% glucose, and 2% fructose, respectively. The growth of yeast cells was observed after three days incubation at 30 °C.

### 4.5. Cloning and Cis-Acting Elements Prediction of LoSWEET14 Promoter

The promoter sequence of *LoSWEET14* was cloned by referring to the instructions of the TAKARA Genome Walking Kit (Takara, Shiga, Japan), and the cis-acting elements on the promoter were predicted on the web of PLACE (http://www.dna.affrc.go.jp/PLACE/, accessed on 10 July 2022). Details of the detected cis-acting elements are listed in Table S1. Additionally, 5′-deletion fragments of the promoter were constructed according to the distribution density of the stress-related cis-acting elements and position of the ABREs, and named *pro1* (−1107 bp), *pro2* (−807 bp), *pro3* (−540 bp), *pro4* (−357 bp), respectively (Figure S4).

### 4.6. Generation of Transgenic Nicotiana tabacum

The ORF of *LoSWEET14* without a stop codon was inserted between the *XhoI* and *SalI* restriction sites of the pSuper1300-GFP vector driven by the CaMV35S promoter, and the full-length promoter and 5′-deletion fragments were inserted between the *HindIII* and *XbaI* restriction sites of the pBI121-GUS vector to replace the original promoter, CaMV35S. The recombinant plasmids and the empty vector were transferred into leaves of *Nicotiana tabacum* ‘NC89′, respectively. Transgenic tobacco seeds were screened with an MS medium containing 30 mg/L Hygromycin. Positive transgenic seedings of the T3 generation were selected for subsequent analysis.

### 4.7. Abiotic Stress Tolerance and ABA Sensitivity Analysis of Transgenic Tobacco

For salt and ABA stress, seeds of WT and *LoSWEET14* transgenic tobacco were sown on an MS medium containing 0 µM ABA/NaCl (CK), 2 µM ABA, 4 µM ABA, and 150 mM NaCl, respectively. The standard sucrose concentration of the MS medium was 3%. Phenotypic changes were observed, and germination rates were measured after 2 weeks. For drought stress, 5-week-old seedings were watered thoroughly, then withheld for 30 days followed by re-watering for 7 days. For low temperature stress, 3-week-old seedings were pretreated at 4 °C for 24 h and then treated at −4 °C for 6 h and −8 °C for 6 h to observe phenotypic changes.

### 4.8. Measurements of Physiological Indexes

Contents of soluble sugar, starch, and sucrose were determined according to the method described in a previous study [40]. Content of soluble protein was determined according to the Total Protein Quantitative Assay Kit (Jiancheng Bioengineering Institute, Nanjing, China); Peroxidase Assay Kit (Jiancheng Bioengineering Institute, Nanjing, China) was used to determine the POD activity.

### 4.9. Histochemical Staining and Expression Analysis of GUS

T3 generation seeds of *LoSWEET14* promoter transgenic tobacco were planted on MS medium for 3 weeks and then were treated with 4 °C for cold treatment, and MS medium containing 50 µM ABA, 200 mM NaCl, and 200 mM mannitol, respectively, for 24 h. The treated leaves were sampled immediately and incubated in GUS reaction buffer (Huayueyang Biotech, Beijing, China) at 37 °C for 24 h in the dark. Then, chlorophyll of the samples was removed with 70% alcohol. The staining of samples was observed under white light of the Leica, Wetzlar, Germany, TL3000 Ergo microscope and photographed [57]. The expression levels of *GUS* gene in *LoSWEET14* promoter transgenic tobacco under different stresses were analyzed as described in Section 4.3.

### 4.10. Yeast One-Hybrid (Y1H) Assay

The Y1H assay was performed referring to the Matchmaker Gold Yeast One-Hybrid System Kit (Clontech, Beijing, China). The ABRE (CACGT) and its mutation ABRE(M) (TGGAT) were inserted between the *Sacl* and *Xhol* restriction sites (upstream of the AbA resistance gene) of the pAbAi vector (bait), respectively. The *LoSWEET14* promoter fragments, pro-LoSWEET14-ABRE1 (−1103 bp to −1000 bp) containing one ABRE element and pro-LoSWEET14-ABRE2 (−499 bp to −334 bp) containing two ABRE elements (Figure S4), were also isolated and inserted into the bait vector, respectively. These recombinant plasmids were linearized and transferred into the Y1H yeast strain and cultured in SD/-Leu plates containing AbA. The full-length of the *LoABF2* gene was inserted into the *EcoRl* and *Xhol* restriction sites of the pGADT7 vector (prey) and co-transformed into positive yeast strains transformed with the recombinant pAbAi vector. The obtained co-transformed yeast strain could grow in SD/-Leu plates containing the corresponding concentration of AbA indicating that LoABF2 is able to bind to this DNA sequence.

### 4.11. Statistical Analysis

In this study, each experiment contained a minimum of three plants per treatment and each value indicated mean ± SE (standard error) of three independent biological replicates. Asterisks indicate a significant difference ** *p* < 0.01 and * *p* < 0.05 compared with the corresponding controls using Student’s *t* test.

### 4.12. Accession Numbers

The coding sequences for *LoSWEET14* (OP298002) and *LoABF2* (OP298003) have been submitted to NCBI.

## Supplementary Materials

The following supporting information can be downloaded at: https://www.mdpi.com/article/10.3390/ijms232315093/s1.

## Abbreviations

ABA	Abscisic acid
SWEET	Sugar will eventually be exported transporter
STs	Sugar transporters
ABRE	ABA-responsive element
GFP	Green fluorescence protein
GUS	β-glucuronidase
TFs	Transcript factors
AbA	Aureobasidin A
TMDs	Transmembrane domains
