# Supplementary material for: LoSWEET14, a Sugar Transporter in Lily, Is Regulated by Transcription Factor LoABF2 to Participate in the ABA Signaling Pathway and Enhance Tolerance to Multiple Abiotic Stresses in Tobacco
Source: Int J Mol Sci. 2022 Dec 1;23(23):15093. doi: 10.3390/ijms232315093 (PMC9739489; doi:10.3390/ijms232315093)
Supplement: Supplementary file 1 [file ijms-23-15093-s001.zip › Figure S1.pdf]

- **>*LoSWEET14* cDNA nucleotide sequence**

- ATGGCCAGGTTATCAATGGACCATCCCTGGGCTTTTGCCTTCGGTATCTTAGGGAACCTCGCGTCATTATT
GGTTTATGTGGCTCCAATCCCAACATTTGTTCGTGTTTATCGGAAGAAATCGACAGAAGGATTCCATTCA
GTTCCGTATGTAGTTGCATTGTTCAGTGCCATGTTGTGGATATACTACGCGTTCATCAAGACAAACTCATA
CCTTCTCATCACCATCAACTCGCTCGGTTGCGTCATCGAAATCGTATACATTATAGTGTTCATAATGTATGC
CTCAAAGAGTGGAAGGGTCCACACTGTTAAGCTAATCTGTGTCATTGACGTTGGACTGTTTGGTCTGATT
GTCCTGGCAACGTACTTCTTCGTAGAGGGAGCCAAACGCTTGACTCTTCTTGGTTGGATCTGTGTTGGG
TTCTCTGTTAGCGTGTTTATTGCCCCACTTAGCATCATGAGGATTGTGATTCGTACAAAGAGTGTCGAGTT
CATGCCATTCTACCTGTCATTCTTTCTCACTCTTAGTGCTGTCACATGGTTCTCCTATGGGCTACTCACCA
AGGATATTTATGTAGCGATCCCGAATACACTGGGTTTCGCATTCGGTGTGTCTCAAATGATACTATATATTA
TCTACATGGACAGGGGACAGAATACCGCAGAGGAGAAAGGAGCAGTGCCAGAGCACATTGTGGACATG
ACAAAAATCGATTTAGCTCCAGATTTTGAGATGAAAGAGTTCCCCGACGTTAAAGAGAAAATGTGTGAA
CTTGTGGTGAAAGGAAGGGCGACACGCGAATTCGATATCGCGGCCGCCTGCAGTCAATACTGA

Figure S1. cDNA nucleotide sequence of *LoSWEET14*
