# Supplementary material for: LoSWEET14, a Sugar Transporter in Lily, Is Regulated by Transcription Factor LoABF2 to Participate in the ABA Signaling Pathway and Enhance Tolerance to Multiple Abiotic Stresses in Tobacco
Source: Int J Mol Sci. 2022 Dec 1;23(23):15093. doi: 10.3390/ijms232315093 (PMC9739489; doi:10.3390/ijms232315093)
Supplement: Supplementary file 1 [file ijms-23-15093-s001.zip › Figure S2.pdf]

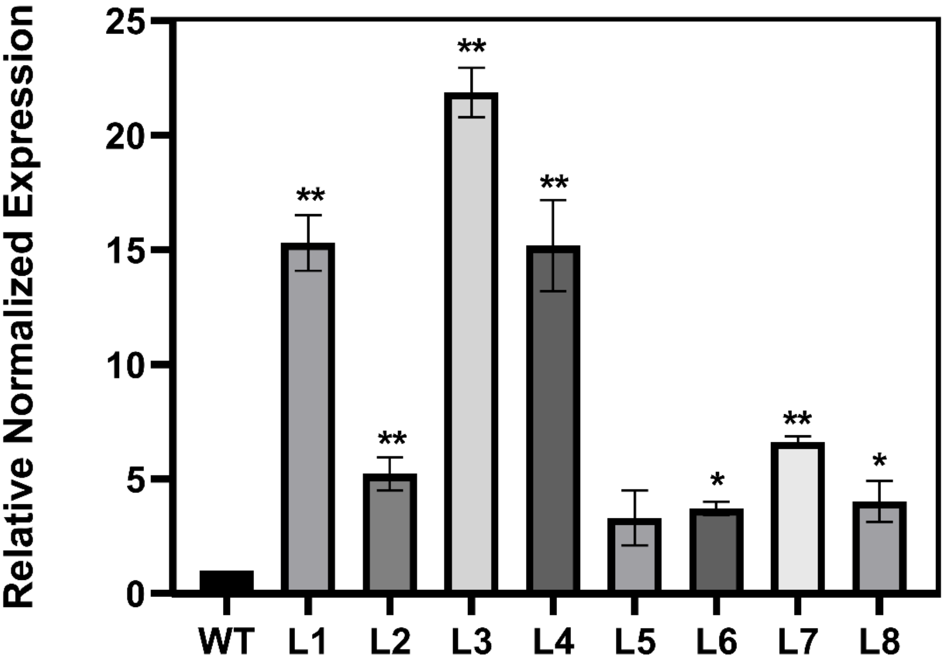

Figure S2. Relative expression of *LoSWEET14* in transgenic lines. Each value indicates Mean ± SE (standard error). Asterisks indicate a significant difference ** $p < 0.01$ and * $p < 0.05$ compared with WT plants.
