# Supplementary material for: LoSWEET14, a Sugar Transporter in Lily, Is Regulated by Transcription Factor LoABF2 to Participate in the ABA Signaling Pathway and Enhance Tolerance to Multiple Abiotic Stresses in Tobacco
Source: Int J Mol Sci. 2022 Dec 1;23(23):15093. doi: 10.3390/ijms232315093 (PMC9739489; doi:10.3390/ijms232315093)
Supplement: Supplementary file 1 [file ijms-23-15093-s001.zip › Figure S4.pdf]

pro1 (-1107bp) *pro-LoSWEET14*-ABRE1

AGTCGAGAGAGATGAAGGAAAGGCCATCGTTGAAGATGCCTCTGCCGACAGTGGTCCCAAAGATGGACCCCCACCCGCGAGGAGCATCGTGG

AAAAAGAAGACGTTTCAACCACGTCTTCAAAGCAAGTGGATTGATGTGATATCTCCACTGACGTAAGGGGTGACGCACAGTCCCACTATCCTTC

GCAAGACCCTTCCTCTATATAAGGAAGTTCATTTCATTTGGAGAGGACCTCGACCTCAACACAACATATACAAAACAAACGAATCTCAAGCAATC

pro2 (-807bp)

AAGCATTCTACTTCTATTGCAGCAATTTAAATCATTTCTTTTAAAGCAAAAGCAATTTTCTGAAAATTTTCACCATTTACGAACGATACTCGAGAT

GGCCAGGTTATCAATGGACCATCCCTGGGCTTTTGCCTTCGGTATCTTAGGGAACCTCGCGTCATTATTGGTTTATGTGGCTCCAATCCCAACATT

pro3 (-540bp)

TGTTCGTGTTTATCGGAAGAAATCGACAGAAGGATTCCATTCAGTTCCGTATGTAGTTGCACTGAACAATGCAACTACATATGGTCGACTGAGAA

pro-LoSWEET14-ABRE2

GAATTTAAGAAACAGAATGACAAGATAGCAATAGTGATGATGTTCGGTATGGAGCCCACAAGAGCCAAAGAGGACGCTAGGCAAAGTGCGCGT

pro4 (-357bp)

GTGTGGATGTGTATCGGCACCAGTCCCAGCATTCAACAGTGTGTCACGTGAAATGAGCCACCTTGCACAACACGTAAGATAAGCTCTCGTCCCT

CAACAGCACACATGTGTGACCCTGATTAATCTCCAAAGGCAAAAACACAGAAGTTATCTTATTGCTGGCTGGAGGAGCAATCATGATATTGCCA

CCTCACACACATGGTAGACAAAATGGAGGGAATGCTCTCCCTGTATAAATATCCGTCTTCCGTTAAAGCTGAAAAACAGACAAATAAGTAAGAG

AGAGAGAGGAGAAGAGAAGAGAAGTAAGTGCGAGAGAAGGAGAGAGGGTCTTAAATACTGTTTTATTTTAATCTTTCTTTGAGGGTGTAATTG

AGAAGGCCACTGTCTTCCCTCTGAACCTAAATATTTTCTTCCATTTTGTACCTCCAAAAGAGGAAGAGAG**ATG**

Figure S4. The promoter sequence of *LoSWEET14*. ABRE elements in this sequence are highlighted in yellow. 5'-deletion fragments of the promoter were constructed and named *pro1* (-1107bp), *pro2* (-807bp), *pro3* (-540bp), *pro4* (-357bp), respectively and the start positions are marked with blue vertical arrows. Fragments used for Y1H assay are marked with brown horizontal arrows.
