# Supplementary material for: LoSWEET14, a Sugar Transporter in Lily, Is Regulated by Transcription Factor LoABF2 to Participate in the ABA Signaling Pathway and Enhance Tolerance to Multiple Abiotic Stresses in Tobacco
Source: Int J Mol Sci. 2022 Dec 1;23(23):15093. doi: 10.3390/ijms232315093 (PMC9739489; doi:10.3390/ijms232315093)
Supplement: Supplementary file 1 [file ijms-23-15093-s001.zip › Figure S6.pdf]

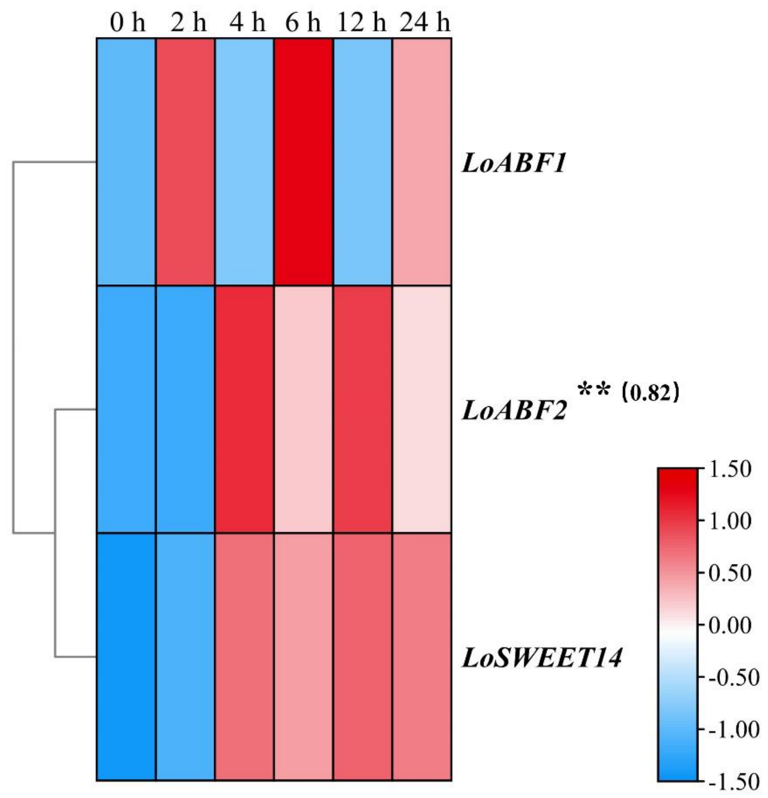

Figure S6. Correlation analysis of expression patterns of *LoSWEET14* and *LoABF* genes under 150 μM ABA treatment. The color scale is displayed on the right side, and the color from red to blue indicates the expression level from high to low. ** showed highly significant at 0.01 probability level.
