# Supplementary material for: LoSWEET14, a Sugar Transporter in Lily, Is Regulated by Transcription Factor LoABF2 to Participate in the ABA Signaling Pathway and Enhance Tolerance to Multiple Abiotic Stresses in Tobacco
Source: Int J Mol Sci. 2022 Dec 1;23(23):15093. doi: 10.3390/ijms232315093 (PMC9739489; doi:10.3390/ijms232315093)
Supplement: Supplementary file 1 [file ijms-23-15093-s001.zip › Figure S7.pdf]

```
1   ATGACGCTGGAGGAGTTCCTAGTGCGGGCCGGGGTCGTGAGGGAG
     M  T  L  E  E  F  L  V  R  A  G  V  V  R  E

46  GATGTGATCAACCAGCAGCCGGCGCTGCCTGCACCTGCGCCGATC
     D  V  I  N  Q  Q  P  A  L  P  A  P  A  P  I

91  TTTGCCGCCACCACTACCACCGGCGCCAACAACAATAATGGCATG
     F  A  A  T  T  T  T  G  A  N  N  N  N  G  M

136 TTCTACAGTGACATGCCCATGCAGTTGCCATCTTCAAGTCGTAAG
     F  Y  S  D  M  P  M  Q  L  P  S  S  S  R  K

181 CAGGGGCTTTCTCTGACATTCAGCCGGCCGGGACCGAGTAACGGA
     Q  G  L  S  L  T  F  S  R  P  G  P  S  N  G

226 TCTGTAATCTCCAACTCAGCAATCTCCAACTCAGGGATGACTAGG
     S  V  I  S  N  S  A  I  S  N  S  G  M  T  R

271 GCGTACCCGGCAGCTGAACTGGTCAGCCCTAAGAGGATGAGGGGT
     A  Y  P  A  A  E  L  V  S  P  K  R  M  R  G

316 GGGGGATTGGTTGGAATGGGTGATACGGCGATGGGAAATGGGTTG
     G  G  L  V  G  M  G  D  T  A  M  G  N  G  L

361 ATGCCCGGCGTGGTTGGAATTGGGGGCGGCGGGGGGATGTCAGCC
     M  P  G  V  V  G  I  G  G  G  G  G  M  S  A

406 GGTGCGTTGGCATCACCGGCGAACCGGATTACCGCGGATGGGTTG
     G  A  L  A  S  P  A  N  R  I  T  A  D  G  L

451 GCAAGGAGTAACGGGGACCTGTCGTCGTTGTCTCCGGTGGCACTG
     A  R  S  N  G  D  L  S  S  L  S  P  V  A  L

496 TTCAATGGAGCGCAGAGAGGGAGGAAAAGTGGGGCAGTGGAGAAA
     F  N  G  A  Q  R  G  R  K  S  G  A  V  E  K

541 GTTGTTGAGAGGAGACAGAAGAGGATGATCAAGAATAGGGAGTCA
     V  V  E  R  R  Q  K  R  M  I  K  N  R  E  S

586 GCAGCCAGATCACGGGCTAGGAAACAGGCATATACGATGGAGCTT
     A  A  R  S  R  A  R  K  Q  A  Y  T  M  E  L

631 GAAGCTGAAATAGCCAAACTTAAAGAGCAGAACGAGGATTTACAG
     E  A  E  I  A  K  L  K  E  Q  N  E  D  L  Q

676 AAAAAGCAGGTGGACATTATGGAGATGCAGAAGAATCAGGTTCTG
     K  K  Q  V  D  I  M  E  M  Q  K  N  Q  V  L

721 GAGGTGATCACCCAGCAGCATGTCCCAAAGAAACGATGCTAA
     E  V  I  T  Q  Q  H  V  P  K  K  R  C  *
```

Figure S7. cDNA nucleotide sequence and amino acid sequence of *LoABF2*.
