# Supplementary material for: LoSWEET14, a Sugar Transporter in Lily, Is Regulated by Transcription Factor LoABF2 to Participate in the ABA Signaling Pathway and Enhance Tolerance to Multiple Abiotic Stresses in Tobacco
Source: Int J Mol Sci. 2022 Dec 1;23(23):15093. doi: 10.3390/ijms232315093 (PMC9739489; doi:10.3390/ijms232315093)
Supplement: Supplementary file 1 [file ijms-23-15093-s001.zip › Figure S8.pdf]

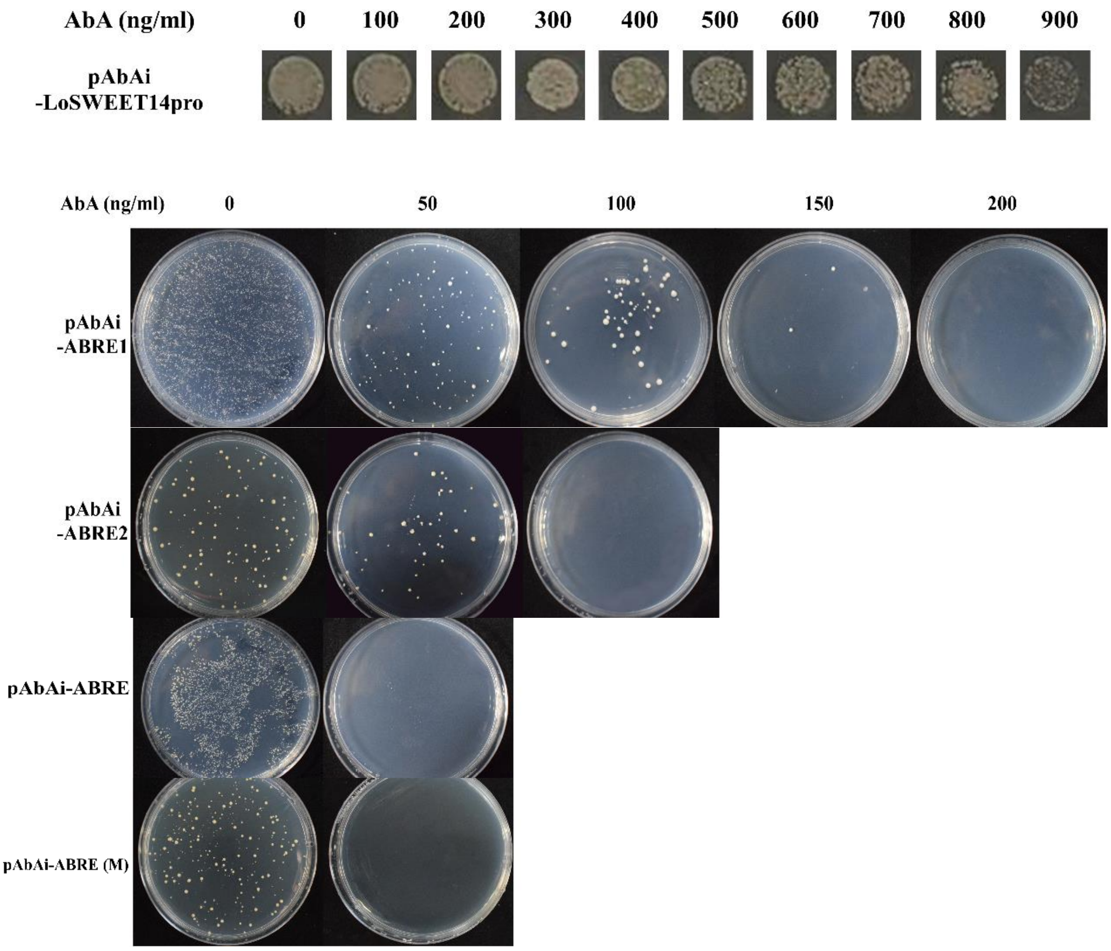
AbA (ng/ml)
0
100
200
300
400
500
600
700
800
900
pAbAi
-LoSWEET14pro
AbA (ng/ml)
0
50
100
150
200
pAbAi
-ABRE1
pAbAi
-ABRE2
pAbAi-ABRE
pAbAi-ABRE (M)

(a)

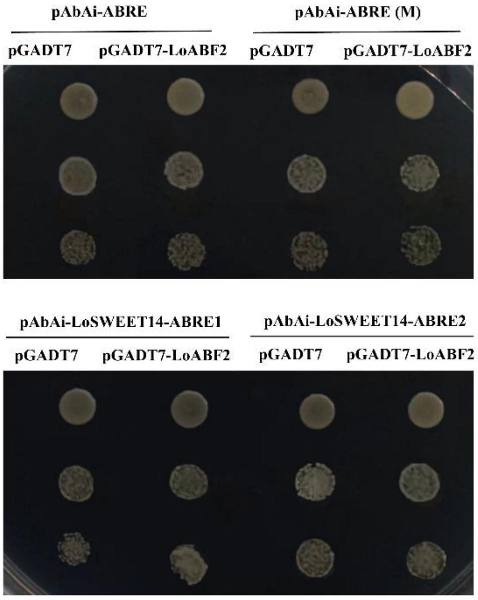
pAbAi-ABRE
pAbAi-ABRE (M)
pGADT7
pGADT7-LoABF2
pGADT7
pGADT7-LoABF2
pAbAi-LoSWEET14-ABRE1
pAbAi-LoSWEET14-ABRE2
pGADT7
pGADT7-LoABF2
pGADT7
pGADT7-LoABF2

(b)

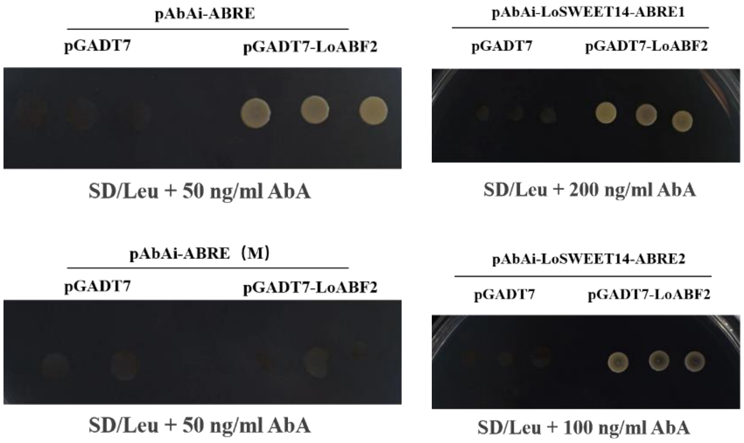

(c)

Figure S8. Growth of yeast strains in Y1H assay. The minimum inhibitory concentrations of AbA for recombinant bait yeast strains (a). Y1H yeast strains were co-transformed into the prey vector pGADT7-LoABF2 with bait pAbAi-ABRE, pAbAi-ABRE(M), pAbAi-LoSWEET14-ABRE1 and pAbAi-LoSWEET14-ABRE2, respectively, and cultured on SD /-Leu medium without AbA (b), and containing AbA with corresponding concentration (c), with pGADT7 empty vector used as the negative control.
