# Supplementary material for: LoSWEET14, a Sugar Transporter in Lily, Is Regulated by Transcription Factor LoABF2 to Participate in the ABA Signaling Pathway and Enhance Tolerance to Multiple Abiotic Stresses in Tobacco
Source: Int J Mol Sci. 2022 Dec 1;23(23):15093. doi: 10.3390/ijms232315093 (PMC9739489; doi:10.3390/ijms232315093)
Supplement: Supplementary file 1 [file ijms-23-15093-s001.zip › Table S1.pdf]

Supplementary Table S1. Stress-related cis-acting elements identified in the promoter region of *LoSWEET14*

time	Sequence	Position (Strands)	Function
DRE core	GCCGAC	43(+)	Core CRT/DRE motif
AuxRR-core	GGTCCAT	62(－), 390(－)	cis-acting regulatory element involved in auxin responsiveness
MYB-binding site	CAACAG	107(+), 694(+), 755(+)	MYB binding site
ABRE	ACGTG	111(－), 705(－), 731(－)	cis-acting element involved in the abscisic acid responsiveness
TGACG-motif	TGACG	150(+), 162(+), 436(－)	cis-acting regulatory element involved in the MeJA-responsiveness
MYC	CATTTG	220(+), 469(+), 765(+), 856(－)	bHLH binding site
ARE	AAACCA	445(－)	cis-acting regulatory element essential for the anaerobic induction
TCT-motif	TCTTAC	734(－), 936(－)	a light responsive element
Box 4	ATTAAT	779(－)	a conserved DNA module involved in light responsiveness
TCCC-motif	TCTCCCT	884(+)	a light responsive element
CAT-box	GCCACT	1041(+)	cis-acting regulatory element related to meristem expression
MRE	AACCTAA	1059(+)	MYB binding site involved in light responsiveness
