# Supplementary material for: LoSWEET14, a Sugar Transporter in Lily, Is Regulated by Transcription Factor LoABF2 to Participate in the ABA Signaling Pathway and Enhance Tolerance to Multiple Abiotic Stresses in Tobacco
Source: Int J Mol Sci. 2022 Dec 1;23(23):15093. doi: 10.3390/ijms232315093 (PMC9739489; doi:10.3390/ijms232315093)
Supplement: Supplementary file 1 [file ijms-23-15093-s001.zip › Table S2.pdf]

Supplementary Table S2 Primers used in this study.

Primers' name	Primer sequence (5'- 3')
qRT-PCR *LoSWEET14*	F: TTCAGTTCCGTATGTAGTTGC
	R: AAGCGTTTGGCTCCCTC
qRT-PCR *LoTIP1*	F: GAAGCCAGAAACGGAGAAGAAT
	R: GTAGGGTGGATTGGGAAGA
qRT-PCR *Ntactin*	F: AATGGAACTGGAATGGTCAAGGC
	R: TGCCAGATCTTCTCCATGTCATCCCA
qRT-PCR *NtABA1*	F: ATTCAGTTCATCCATCCACTTCA
	R: ACTAACCCTCCAATCCCACC
qRT-PCR *NtABI1*	F: ACGAATGCCTTATTCTTGCG
	R: TTATGTTGTCCTTGCTGCCC
qRT-PCR *NtABI2*	F: TGCCATTGTTTGTTCATCTCAC
	R: GGAATAATCCAAGGCTTCAAGTATC
qRT-PCR *NtAPX1*	F: GAGAAATATGCTGCGGATGA
	R: CGTCTAATAACAGCTGCCAA
qRT-PCR *NtCAT*	F: AGGTACCGCTCATTCACACC
	R: AAGCAAGCTTTTGACCCAGA
qRT-PCR *NtCAX3*	F: TCATTGCTGTCCCTTTTGCC
	R: CAGTTCCGTTGCGTTCCC
qRT-PCR *NtERD10c*	F: AACGTGGAGGCTACAGATCG
	R: GTTCCTCTTGGGCATGAGTT
qRT-PCR *NtSOS1*	F: GGAAAGTTTTGAATTGCCTCAC
	R: GAGTGTGATAATAGCGAAGACGAT
qRT-PCR *GUS*	F: GGGCAACAAGCCGAAAGA
	R: GCCAGTGGCGCGAAATAT
qRT-PCR *LoABF1*	F: TTGGAATGGGCGATACGG
	R: CGGAGACAACGACGACAGG
qRT-PCR *LoABF2*	F: ACCTTTCGTCGCTTTCCC
	R: CTGCCTCCTTTCAACCACTT
LoSWEET14 CDS	F: ATGGCAGGGTTATCAATGGA
	R: CCTTCACATCAAGTTCACAC
LoABF2 CDS	F: ATGACGCTGGAGGAGTTCC
	R: TTAGCATCGTTTCTTTGGGA
LoSWEET14 Promoter	SP1: TCTCTTTCTCTCGCACTTACTTCC
	SP2: ACACTGTTGAATGCTGGGACTGGT
	SP3: TAGCGTCCTCTTTGGCTCTTGTAG
pBI121-*pro1*-GUS	F: GACCATGATTACGCCAAGCTT(*Hind*III)AGTCGAGAGAGATGAAGGAAAGG
	R: ACCACCCGGGGATCCTCTAGA (*Xba*I)CTCTCTTCCTCTTTTGGAGGTACAA
pBI121-*pro2*-GUS	F: GACCATGATTACGCCAAGCTT(*Hind*III)GCAGCAATTTAAATCATTTCTTTTA
	R: ACCACCCGGGGATCCTCTAGA (*Xba*I)CTCTCTTCCTCTTTTGGAGGTACAA
pBI121-*pro3*-GUS	F: GACCATGATTACGCCAAGCTT(*Hind*III)TGTCGACTGAGAAGAATTTAAGAA
	R: ACCACCCGGGGATCCTCTAGA (*Xba*I)CTCTCTTCCTCTTTTGGAGGTACAA

pBI121-*pro4*-GUS	F: GACCATGATTACGCCAAGCTT(*Hind*III)GATAAGCTCTCGTCCCTCAACAG
	R: ACCACCCGGGGATCCTCTAGA (*Xba*I)CTCTCTTCCTCTTTTGGAGGTACAA
pDR196- *LoSWEET14*	F: TATACCCCAGCCTCGACTAGT(*Spe*I)ATGGCCAGGTTATCAATGGACC
	R: GATAAGCTTGATATCGAATTC(*EcoR*I)GTATTGACTGCAGGCGGCC
pAbAi-*LoSWEET14*-ABRE1	F: AAGCTTGAATTCGAGCTC(*Sac*I)GCGAGGAGCATCGTGGAAA
	R: ATACAGAGCACATGCCTCGAG(*Xho*I)AGTGGGACTGTGCGTCACCC
pAbAi-*LoSWEET14*-ABRE2	F: AAGCTTGAATTCGAGCTC(*Sac*I)ATGTTCGGTATGGAGCCCACA
	R: ATACAGAGCACATGCCTCGAG(*Xho*I)CACATGTGTGCTGTTGAGGGAC
pAbAi-ABRE	F: AGCTACGTGACGTGACGTGTCGA
	R: CACGTCACGTCACGT
pAbAi-ABRE(M)	F: AGCTTGGATTGGATTGGATTCGA
	R: ATCCAATCCAATCCA
pGBKT7-*LoABF2*	F: GCCATGGAGGCCAGTGAATTC(*EcoR*I)ATGAACTACAAGAACATGGGCTCG
	R: ACGATTCATCTGCAGCTCGAG(*Xho*I)GCATCGTTTCTTTGGGACATG
